# A multimodal machine learning system in early screening for toddlers with autism spectrum disorders based on the response to name

**DOI:** 10.3389/fpsyt.2023.1039293

**Published:** 2023-01-26

**Authors:** Feng-lei Zhu, Shi-huan Wang, Wen-bo Liu, Hui-lin Zhu, Ming Li, Xiao-bing Zou

**Affiliations:** ^1^Child Developmental and Behavioral Center, The Third Affiliated Hospital of Sun Yat-sen University, Guangzhou, China; ^2^School of Electronics and Information Technology, Guangzhou Higher Education Mega Center, Sun Yat-sen University, Guangzhou, China; ^3^Data Science Research Center, Duke Kunshan University, Kunshan, China

**Keywords:** autism spectrum disorder (ASD), response to name (RTN), multimodal, machine learning, early screening, identification

## Abstract

**Background:**

Reduced or absence of the response to name (RTN) has been widely reported as an early specific indicator for autism spectrum disorder (ASD), while few studies have quantified the RTN of toddlers with ASD in an automatic way. The present study aims to apply a multimodal machine learning system (MMLS) in early screening for toddlers with ASD based on the RTN.

**Methods:**

A total of 125 toddlers were recruited, including ASD (*n* = 61), developmental delay (DD, *n* = 31), and typical developmental (TD, *n* = 33). Procedures of RTN were, respectively, performed by the evaluator and caregiver. Behavioral data were collected by eight-definition tripod-mounted cameras and coded by the MMLS. Response score, response time, and response duration time were accurately calculated to evaluate RTN.

**Results:**

Total accuracy of RTN scores rated by computers was 0.92. In both evaluator and caregiver procedures, toddlers with ASD had significant differences in response score, response time, and response duration time, compared to toddlers with DD and TD (all *P*-values < 0.05). The area under the curve (AUC) was 0.81 for the computer-rated results, and the AUC was 0.91 for the human-rated results. The accuracy in the identification of ASD based on the computer- and human-rated results was, respectively, 74.8 and 82.9%. There was a significant difference between the AUC of the human-rated results and computer-rated results (*Z* = 2.71, *P*-value = 0.007).

**Conclusion:**

The multimodal machine learning system can accurately quantify behaviors in RTN procedures and may effectively distinguish toddlers with ASD from the non-ASD group. This novel system may provide a low-cost approach to early screening and identifying toddlers with ASD. However, machine learning is not as accurate as a human observer, and the detection of a single symptom like RTN is not sufficient enough to detect ASD.

## 1. Introduction

Autism spectrum disorder (ASD) is a set of heterogeneous neurodevelopmental conditions, characterized by early onset deficits in social communication, restricted interest, and repetitive behaviors (1). The prevalence of ASD has sustainably increased in recent decades. The latest epidemiological survey has reported that approximately one in 44 children (2.27%) was identified as having ASD in the United States (2). Previous studies have shown that the symptoms of ASD emerge at an early age (3), while the diagnostic age of ASD exists a significant delay. An investigation has demonstrated that the mean age at ASD diagnosis was 4.4 years, whereas the mean diagnostic delay was 2.2 years (4). Initiating early intervention can improve the prognosis of ASD (5), and the diagnostic delay implies the loss of the opportunity to receive early intervention for toddlers with ASD.

Response to name (RTN) is a crucial social response for toddlers. RTN plays an essential recognition functioning in social cognition and interaction. Toddlers aged 4–6 months listen significantly longer to their own names than others, suggesting RTN is an internalized behavior early in life (6). Toddlers with typical development (TD) significantly prefer their own names to others, especially those spoken by their mothers (7). Due to the crucial behavior in the development of social skills, the assessment of RTN may be useful in early screening and identification of ASD.

Many lines of evidence have shown a reduced RTN in toddlers with ASD (8, 9). A prospective study has examined the longitudinal patterns of RTN from 6 to 24 months and found that toddlers who consistently fail to respond to their names in the second year of life may be at risk not only for ASD but also for greater impairment in later age (10). The results suggest that persistent failure to RTN in early life may be a critical indicator for developmental disorders. Many screening and diagnostic instruments have included RTN as an important item (11, 12), such as the modified checklist for autism in toddlers (M-CHAT) and the autism diagnostic observation schedule (ADOS). Retrospective studies have found that diminished RTN relies merely on the parent’s report or schedule (13, 14), whereas little is known to quantize RTN *via* the more accurate method.

The existing methods in screening ASD mainly rely on scale screening, such as M-CHAT and social communication questionnaire (SCQ) by parent’s report, with some subjective errors and long time-consuming. Compared with the disadvantages of traditional methods, machine learning has risen to be a promising alternative in the screening and diagnosis of ASD (15, 16). Machine learning aims to construct predictive models from the datasets, which encompasses search methods, artificial intelligence, and mathematical modeling. Machine learning algorithms are applied as an intelligent method with minimal human involvement, with decision tree algorithms being used in data processing to detect ASD. A study has applied machine learning to distinguish ASD from TD children using a simple upper-limb reach-to-drop task (17), and the resulting model showed an accuracy rate of 96.7%, which suggest that machine learning could be a useful method of classification and discrimination in the diagnosis of ASD. Recently, Achenie et al. have demonstrated that the machine learning method was comparable to the M-CHAT with follow-up items in the accuracy of ASD diagnosis using fewer items (18). The results suggest that machine learning may be a promising tool in implementing automatic, efficient scoring in the diagnosis of ASD. A multimodal machine learning system is a more advanced machine learning, which is a data-driven approach for early autism screening that combines general machine learning principles and behavioral analysis techniques to perform comprehensive computer analysis of multimodal audio–visual data. By extracting useful information and building complex models that surpass human performance in analyzing large datasets (19), MMLS can enhance our understanding of ASD and may further build a stronger foundation for early screening and identification.

To the best of our knowledge, toddlers with ASD are still mainly diagnosed by clinicians taking a long time to collect behavioral observations and case history. The early screening and identification of ASD are also major challenges to clinicians. Little is known about the question of whether MMLS could enhance or replace the role of clinicians in the screening and identification of ASD, and few studies have investigated the difference in RTN between ASD and non-ASD groups. The primary objective of our study was to explore the difference in RTN between toddlers with ASD and non-ASD groups using the MMLS. We aim to test the feasibility of the application of the machine approach in the rating works of RTN to predict ASD.

## 2. Materials and methods

### 2.1. Participants

We designed developmental delay (DD) and typical development (TD) as non-ASD groups. Toddlers with ASD and DD were randomly recruited from the inpatients or outpatients in Child Developmental and Behavioral Center, the Third Affiliated Hospital of Sun Yat-sen University in Guangzhou between April and November 2017. Meanwhile, we recruited toddlers with TD from the nearby community during the same period. Toddlers with ASD (*n* = 61) were matched on chronological age to toddlers with DD (*n* = 31) and TD (*n* = 33), aged 16–30 months. The chronological age of children with ASD was 25.16 (3.71) months, and the sex ratio of men to women was 5.1:1. The chronological age of children with DD was 25.09 (3.68) months, and the sex ratio in this group was 5.2:1. The chronological age of children with TD was 24.73 (3.37) months, and the sex ratio was 5.6:1. There were no significant differences in chronological age and sex ratio among the three groups (all *P*-values > 0.05).

Toddlers with ASD were included to meet the diagnostic criteria of the diagnostic and statistical manual of mental disorders-the 5th edition (DSM-5) (20). Exclusionary criteria for toddlers with ASD included the following: (1) symptomatic autism, such as Rett syndrome, Fragile X syndrome, Angelman syndrome, and Prader–Willi syndrome, which were caused by known genetic defects or inherited metabolic diseases; (2) hearing impairment and moderate-to-severe sensory impairment; (3) cerebral palsy and poorly controlled seizures. The diagnosis of ASD in this study required a coincident diagnosis by two clinicians to ensure the quality and validity of the diagnosis.

Inclusion criteria for toddlers with DD included the following: (1) isolated developmental delay (involving single domain); (2) multiple developmental delays—two or more domains or developmental lines affected; (3) global developmental delay (GDD)—significant delay in most of the developmental domains (21). Exclusionary criteria for toddlers with DD included the following: (1) physical disabilities; (2) hearing impairment; (3) a history of serious brain diseases at birth, such as hypoxic ischemic encephalopathy and neonatal apoplexy.

Toddlers with TD were assessed by professional staff before the study, which did not have any confirmed or suspected developmental disorders in healthcare facilities. Exclusion criteria for toddlers with TD were as follows: (1) hearing impairment; (2) visual impairment; (3) speech and vocal impairment; (4) motor impairment due to any physical disability.

### 2.2. Procedures

**FIGURE 1:**
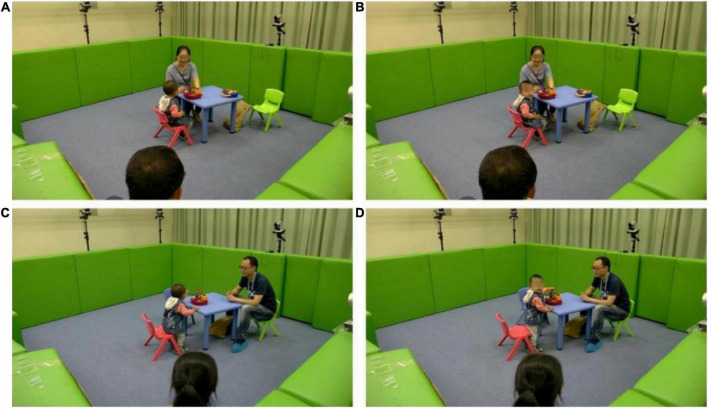
Demonstration of different name call procedures. **(A)** Before the name call of the evaluator procedure. **(B)** After the name call of the evaluator procedure. **(C)** Before the name call of the caregiver procedure. **(D)** After the name call of the caregiver procedure.

To avoid habituation during frequent name calls, the evaluator or caregiver was requested to call the toddler’s name no more than three times. The place of name call was chosen about 2 m behind the toddler according to the social distance (22). The evaluator first explained the experimental process and instructions to the caregiver and then guided the toddler going into the laboratory with a standard demonstration. The study began with toddlers playing with the preparative toy (jack in the box) placed on the table, with the caregiver sitting beside it. When the toddler’s attention was completely attracted by the toy, the evaluator began to call his/her name behind. If the toddler did not respond to the initial name call, the evaluator paused for approximately 3 s and then called the toddler’s name again. In this progress, up to three name calls were implemented until the toddler turned their head with eye contact or there was no response. The toddler was next allowed to freely play for 30 s. When the toddler’s attention was focused on the toy again, the caregiver called the toddler’s name by repeating the above-mentioned procedures, and the same is illustrated in Figure 1.

### 2.3. Automatic name calling detection

**FIGURE 2:**
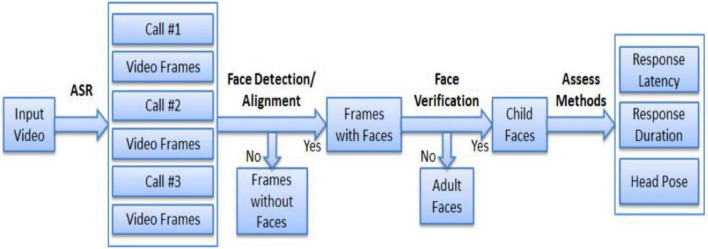
Proposed multimodal machine learning framework toward RTN. *ASR, automatic speech recognition; RTN, response to name.

An overview of the proposed multimodal “response to name” assessment framework is shown in Figure 2. In our study, both speech processing and vision-based methods are incorporated to minimize human annotations in assessing responsiveness. In particular, we simultaneously consider response speed, response duration, and head and pose information to jointly determine a predicted score. A core task in “response to name” experiments is to locate the time stamps of name callings such that response latency can be measured. Name calling is annotated manually and incorporates unnecessary human interaction.

### 2.4. Data collection and processing

Behavioral data were obtained from eight high-resolution cameras which were evenly distributed around the laboratory. The voice was recorded by a digital audio recorder worn by the toddlers. The sensors are synchronized and are expected to comprehensively cover various modalities of the signals from the child. The assessment framework is illustrated in Figure 2.

#### 2.4.1. Clinical scoring

For the manual scoring method, the videos were played frame by frame through a free video player (the KM player). Every second of the video contains 30 frames. Items are scored from 0 to 2: “0” means the toddler had no response; “1” means the toddler turned their head toward the evaluator but without eye contact; “2” stands for the toddler turned their head toward the evaluator and had eye contact with the evaluator.

Response time (RT) means the latency from the time which caller’s voice was detected to the time which toddler’s face was identified. If no response, we defined a maximal value of 10 s as RT. Response duration time (RDT) represents the time of video segments that contain the toddler’s face. If no response, the RDT is 0. Only the response that appeared behind the name calling was viewed as valid. Furthermore, to compare the results of manual scoring and machine learning, two co-investigators and an expert in the diagnosis of ASD reviewed these videos, who were blind to the diagnosis and current group membership.

#### 2.4.2. Automatic name calling detection

A core step of data processing was to locate the time stamp of the name calling. We designed an automatic speech recognition (ASR) system based on Kaldi, a toolbox widely used for speech recognition (23). In the registration step, each child’s name was registered in the system. In addition, the ASR feature was matched to the system to obtain the time stamp during the name calling.

#### 2.4.3. Face detection and recognition

Another critical step was to locate the child’s face in the video. In this study, we used the DLib implementation of the face alignment methods proposed by Kazemi et al. to detect and align the faces simultaneously (24). Besides detecting faces, the algorithm returned 68 landmarks which later would be used to compute the head pose. Meanwhile, we also performed deep learning-based face recognition to identify a child’s face. Similar to name calling detection, we registered each child’s face in the system and verified every detected face based on the method.

#### 2.4.4. Feature representation

With the detected child faces, we proposed a rule-based “response to name” assessment framework. Consisting with conventional clinical diagnosis, the framework was based on the following basic assumptions: The first assumption was that a clear response should happen with a relatively small latency upon calling. The second was that a clear response should last for a certain length of duration.

The response latency in the first assumption can be naturally modeled as follows:


$$\text{Latency}={\text{T}}_{\text{f}}-{\text{T}}_{\text{c1}}$$

T_*f*_ and T_*c*1_ indicate the time stamp of the first detected face and the beginning of the first call, respectively.

The response duration in the second assumption can also be modeled as follows:


$$\text{Duration}={\text{N}}_{\text{f}}/\text{Frame rate}$$

Nf represents the total number of frames containing the detected child faces.

#### 2.4.5. Score prediction

The data were divided into training and testing sets with a 10-fold cross-validation strategy, and then, we evaluated each subject in an iterative manner.

In this task, a positive response should have a relatively short response latency and a long duration of eye contact. In other words, a typically developing test subject should respond to his name quickly and face the name caller for some time. We thus used response latency and front-facing duration as our features and applied the rule-based decision tree separately on each feature. Besides regressing to the clinical score, we also evaluated the performance of machine evaluation scores in direct ASD prediction. In our test protocol, the parent was first asked to participate in the name calling, followed by the doctor. Thus, there were two responses to name tests performed on each test subject. The ASD prediction result was jointly learned by these two tests.

### 2.5. Statistical analysis

Data management and analysis were performed by the statistical package for social sciences (version 20.0; SPSS Inc., Chicago, IL, USA). For quantitative data, normality tests were first performed. Data that followed the normal distribution were statistically described by the mean and standard deviation (SD), and *t*-tests were applied to data comparison. Data that did not follow the normal distribution are described by the median (M) and interquartile range (IQR). Before group comparisons, the Kruskal–Wallis test was used to estimate the overall distribution. If the overall distributions were not identical, a multisample Kruskal–Wallis one-way ANOVA test by ranks was used to perform multiple comparisons between groups. Both quantitative and qualitative analyses were utilized to examine the current data. The quantitative analysis comprised independent samples *t*-tests and non-parametric tests. Qualitative analyses constituted the chi-square test. The difference in gender across groups was examined by the chi-square test. The difference in chronological age between the groups was examined using independent samples *t*-tests. Internal consistency reliability between two co-investigators was tested by the method of Cronbach. Receiver operating characteristic (ROC) analysis was used in predicting the diagnostic accuracy in toddlers with ASD. MedCalc 15.8 statistical software was applied to compare two ROC curves. In addition, accuracy, specificity, and sensitivity were conducted as an evaluation index in machine learning. A *P*-value of < 0.05 was considered statistically significant.

## 3. Results

### 3.1. Consistency of RTN scores between the human rating and computer rating

Two colleagues rated the video data, respectively, and Cronbach’s alpha between colleagues was 0.98. Assessing results by human rating were used as the standard score. In the evaluator procedure, there were, respectively, 61 valid responses in ASD, 31 in DD, and 33 in TD. In the caregiver procedure, the valid responses were, respectively, 60 in ASD, 31 in DD, and 32 in TD. Taken together, the total consistency of the computer rating was 0.92 in these 248 valid responses as shown in Table 1.

**TABLE 1:** Consistency of human and computer rating.

Item	Score	Evaluator procedure		Caregiver procedure	
		Human	Computer	Human	Computer
ASD		*n* = 61		*n* = 60	
	0	37 (60.66%)	36 (59.02%)	30 (50.00%)	27 (45.00%)
	1	2 (3.28%)	0 (0.00%)	3 (5.00%)	0 (0.00%)
	2	22 (36.07%)	22 (36.07%)	27 (45.00%)	25 (41.67%)
	Consistency	0.95		0.87	
DD		*n* = 31		*n* = 31	
	0	6 (19.36%)	6 (19.36%)	5 (16.13%)	4 (12.90%)
	1	2 (6.45%)	0 (0.00%)	1 (3.22%)	0 (0.00%)
	2	23 (74.19%)	21 (67.74%)	25 (80.65%)	24 (77.42%)
	Consistency	0.87		0.90	
TD		*n* = 33		*n* = 32	
	0	6 (18.18%)	6 (18.18%)	4 (12.50%)	4 (12.50%)
	1	0 (0.00%)	0 (0.00%)	0 (0.00%)	0 (0.00%)
	2	27 (81.82%)	27 (81.82%)	28 (87.50%)	27 (84.38%)
	Consistency	1.00		0.97	
	Total consistency	0.92			

ASD, autism spectrum disorder; DD, developmental delay; TD, typical development.

### 3.2. Comparison of RTN indicators among ASD, DD, and TD

To study the response characteristics in different toddlers, response score, response time, and response duration time were evaluated. Both in the evaluator procedure and the caregiver procedure, there were significant differences in the three indicators between ASD and TD, ASD, and DD (all *P*-values < 0.05), while the difference in the three indicators between DD and TD was not significant (all *P*-values > 0.05). The results are shown in Table 2.

**TABLE 2:** Computer-rated results of RTN among toddlers with ASD, DD, and TD.

Items	ASD	DD	TD	H value	*P*-value	Multiple comparison	Standard H value	Revised *P*-value
	M (IQR)	M (IQR)	M (IQR)					
**Evaluator procedure**								
Score	0	2	2	20.27	<0.001	ASD-TD	-4.12	<0.001
	(2)	(2)	(0)			ASD-DD	-3.10	0.006
						DD-TD	-0.75	1.000
Response time	10	3	2.17	25.98	<0.001	ASD-TD	4.51	<0.001
	(4.17)	(8.14)	(5.10)			ASD-DD	3.78	<0.001
						DD-TD	0.57	1.000
Duration time	0	1.88	1.73	26.53	<0.001	ASD-TD	-4.33	<0.001
	(1.22)	(2.31)	(2.52)			ASD-DD	-4.10	<0.001
						DD-TD	-0.13	1.000
**Caregiver procedure**								
Score	1	2	2	17.08	<0.001	ASD-TD	-3.35	0.002
	(2)	(0)	(0)			ASD-DD	-3.41	0.002
						DD-TD	0.15	1.000
Response time	9.05	2.00	1.80	19.14	<0.001	ASD-TD	3.69	0.001
	(7.31)	(3.59)	(6.82)			ASD-DD	3.46	0.002
						DD-TD	0.10	1.000
Duration time	0.10	1	1.45	10.90	0.004	ASD-TD	-2.84	0.013
	(1.48)	(2.04)	(1.52)			ASD-DD	-2.54	0.033
						DD-TD	-0.19	1.000

ASD, autism spectrum disorder; DD, developmental delay; TD, typical development. Revised *P*-value, *P*-value after Bonferroni correction; standard *H*-value, *H*-value after Bonferroni correction.

### 3.4. ASD diagnostic efficiency, sensitivity, specificity, and accuracy based on the computer- and human-rated results of RTN

**FIGURE 3:**
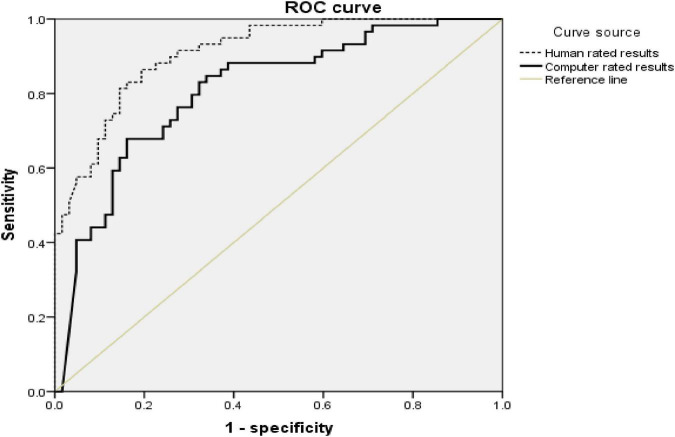
ROC curves of ASD diagnosis from all sample toddlers by computer- and human-rated results.

To evaluate the diagnostic efficiency of using computer rating results to detect ASD, ROC curve (Figure 3) was established for all data of RTN. We combined toddlers with DD and TD as a non-ASD group. Taking the results of both procedures into account, the area under the curve (AUC) was 0.81 (Table 3). The sensitivity, specificity, and accuracy of the ASD diagnosis based on the computer-rated results of RTN were, respectively, 80.0, 69.8, and 74.8% (Table 4). For human-rated results, the AUC was 0.91, and the sensitivity, specificity, and accuracy were, respectively, 83.3, 82.5, and 82.9% (Table 5).

**TABLE 3:** Area under the ROC curves of ASD diagnosis from toddlers with ASD, DD, and TD based on the human- and computer-rated RTN results.

Test variable	Area	Standard error	*P*-value	95% confidence interval	
				Lower limit	Upper limit
Computer rated results	0.81	0.03	<0.001	0.86	0.96
Human rated results	0.91	0.04	<0.001	0.73	0.89

**TABLE 4:** Outcomes of ASD diagnosis based on the computer-rated results of RTN.

Item		Clinical diagnosis		Total
		ASD (*n*)	Non-ASD (*n*)	
Computer diagnosis	ASD	48	19	67
	Non-ASD	12	44	56
	Total	60	63	123

ASD, autism spectrum disorder; Non-ASD, typical developmental and developmental delays.

**TABLE 5:** Outcomes of ASD diagnosis based on the human-rated results of RTN.

Item		Clinical diagnosis		Total
		ASD (*n*)	Non-ASD (*n*)	
Human diagnosis	ASD	50	11	61
	Non-ASD	10	52	62
	Total	60	63	123

ASD, autism spectrum disorder; Non-ASD, typical developmental and developmental delays.

MedCalc 15.8 statistical software was used to compare the AUC of different ROC curves. There was a significant difference between the AUC of the human-rated results and computer-rated results (*Z* = 2.71, *P*-value = 0.007). The difference in accuracy between the computer diagnosis and human diagnosis was not significant (χ^2^ = 2.44, *P*-value = 0.118).

## 4. Discussion

This study began with the aim of assessing the application of artificial intelligence in the early screening of children with ASD. The results indicate that the computer system can perform as effectively as humans in behavior coding. The present study investigated the early social skills of RTN among toddlers with ASD, TD, and TD. Compared with the non-ASD group, toddlers with ASD showed a reduced RTN call. Participants have rated automatically, which provides an advantage over previous screening methods to overcome the inaccuracy of manual scoring of video-based studies.

The total accuracy (92%) of the computer system was relatively high, which indicates that MMLS can accurately reflect the actual performance of different toddlers in RTN procedures. Among these toddlers, accuracy for toddlers with TD was the highest, especially in the evaluator’s procedure. This phenomenon can be attributed to toddlers with TD being more easily adjusted to the new environment and more cooperative compared with the other two groups. Thus, their behavioral information was more easily and accurately captured. Nevertheless, toddlers with ASD had a tendency to perform hyperactively and exhibited more uncontrollable behaviors, which might make it more difficult to quantify behavioral data. Compared with the previous studies on the automatic detection of RTN, the current study used multimodal information to evaluate behavioral data, making the results more reliable.

Our results demonstrated that toddlers with ASD were more likely to fail to respond to name calls compared with the non-ASD group. A recent study has revealed that children with ASD responded to their names significantly less frequently than children without ASD using computer vision analysis (CVA). The CVA also exhibited that children with ASD who did orient to name call showed a longer latency before turning their head (25), which is consistent with our results. The diminished response can be explained by the theory of social motivation and social cognition in ASD. According to the social motivation theory, ASD was an extreme example of reduced social motivation (26). Decreased social orienting and social reward may be the main reason for the low response rate and long response latency to name call, and diminished social maintenance may be the main cause of shorter response duration time in toddlers with ASD. Moreover, restricted patterns of interest in toddlers with ASD may further impact their interest in social stimulations, such as name call. Evidence has manifested that social cognition was positively associated with social functioning (27). Social cognition deficits may cause difficulties in toddlers with ASD to understand societal situations. As a result, toddlers with ASD did not realize that they should give a response to the name call and they did not know what kinds of response they should give, or they did not even understand that the name call was pointed to them. Other possible explanations for the poor response may be that toddlers with ASD have deficits in the ability of auditory spatial attention (28). Studies in adults with ASD also suggested that atypical auditory object processing had implications in understanding the communication difficulties of ASD (29).

Delays in identification and diagnosis have direct bad consequences in timely intervention for children with ASD. The later the initiating intervention, the less opportunity for toddlers with ASD to reach optimal outcomes. This machine learning provides a new approach to screening and identifying ASD, as it is able to identify toddlers with ASD at 2 years old. Furthermore, this machine learning is not difficult to carry out, without any harmfulness. The coding course is replaced by computers, reducing the difference in the evaluator’s subjectivity. The present study showed that the difference in accuracy in the diagnosis of ASD between the computer and humans was not significant. The above-mentioned results suggest that computers and humans had similar performance in behavior coding. This finding has significant implications for developing an artificial intelligence test system in the early screening and diagnosing of ASD. The existing method in screening and prediction of ASD mainly relies on a scale screening and an empirical diagnosis by clinicians, which lacks objective tools. The current study provides a novel approach to coding video-based behavioral data objectively and intelligently, which may offer a complementary method for reliably and effectively assessing toddler behavior.

As a standardized paradigm, the present study has examined the feasibility of early screening of ASD using machine learning. In this approach, MMLS may save human resources in screening and diagnosing ASD. Moreover, it may provide a chance to be earlier identified for toddlers who have suffered a long waiting time before a comprehensive assessment in areas where medical resources are relatively deficient. There are still several limitations in our study. First, the current research is a cross-sectional study, while the development of social abilities in toddlers is a dynamic process. The social performance of one time point cannot reflect the whole societal ability. Second, our rating system could not completely discern all behavioral data as the experiment was conducted in a semi-structure environment and the toddlers must sit in a designated position, which might influence the behavioral performance of toddlers in a natural situation. Third, RTN cannot be used as a single indicator to predict ASD. The next step in future research ought to design a prospective method, including larger sample size and longitudinal follow-up. Moreover, machine learning is not as accurate as a human observer and the detection of a single symptom like RTN is not sufficient enough to detect ASD. The role of multiple social indicators of ASD should be involved in the future study. In addition, the procedure of RTN should be carried out under a free-play situation, where behaviors would be more natural and in line with ecological validity.

## 5. Conclusion

In summary, our results confirm that MMLS can accurately quantify behaviors in RTN procedures and effectively distinguish toddlers with ASD from the non-ASD group. This novel system may provide an accurate and low-cost approach to early screening and identifying toddlers with ASD.

## Data availability statement

The raw data supporting the conclusions of this article will be made available by the authors, without undue reservation.

## Ethics statement

The studies involving human participants were reviewed and approved by The Third Affiliated Hospital of Sun Yat-sen University. Written informed consent to participate in this study was provided by the participants’ legal guardian/next of kin. Written informed consent was obtained from the individual(s), and minor(s)’ legal guardian/next of kin, for the publication of any potentially identifiable images or data included in this article.

## Author contributions

F-LZ: conceptualization and methodology. S-HW: data curation and original—draft preparation. W-BL: visualization and investigation. H-LZ: software and supervision. ML: software and validation. X-BZ: reviewing and approval publication. All authors critically reviewed the manuscript drafts and approved the final manuscript.
